# The Association of Oxidative Stress Status with Open-Angle Glaucoma and Exfoliation Glaucoma: A Systematic Review and Meta-Analysis

**DOI:** 10.1155/2019/1803619

**Published:** 2019-01-15

**Authors:** Binghua Tang, Shengjie Li, Wenjun Cao, Xinghuai Sun

**Affiliations:** ^1^Department of Clinical Laboratory, Eye & ENT Hospital, Shanghai Medical College, Fudan University, Shanghai, China; ^2^Department of Ophthalmology & Visual Science, Eye & ENT Hospital, Shanghai Medical College, Fudan University, Shanghai, China; ^3^State Key Laboratory of Medical Neurobiology, Institutes of Brain Science, Fudan University, Shanghai, China; ^4^Key Laboratory of Myopia, Ministry of Health, Fudan University, Shanghai, China; ^5^Shanghai Key Laboratory of Visual Impairment and Restoration, Fudan University, Shanghai, China

## Abstract

**Purpose:**

To systematically evaluate the associations between oxidative stress status and different types of glaucoma.

**Design:**

Systematic review and meta-analysis.

**Methods:**

We searched PubMed, EMBASE, and the Web of Science for randomized controlled trials written in the English language between January 1, 1990, and November 30, 2016. A random effects model was used to estimate oxidative stress status along with weighted mean differences and 95% confidence intervals (CIs). A funnel plot analysis and Egger's test were performed to assess potential publication bias.

**Main outcome measures:**

Oxidative stress status was abnormal and different in patients with OAG (open-angle glaucoma) and EXG (exfoliation glaucoma).

**Results:**

Blood TAS (total antioxidant status) was lower in the OAG group than in the control group, with a mean difference of 0.580 mmol/L (*p* < 0.0001, 95% CI = −0.668 to −0.492). The aqueous humor SOD (superoxide dismutase), GPX (glutathione peroxidase), and CAT (catalase) levels were higher in the OAG group than in the control group, with mean differences of 17.989 U/mL (*p* < 0.0001, 95% CI = 14.579–21.298), 12.441 U/mL (*p* < 0.0001, 95% CI = 10.423–14.459), and 1.229 fmol/mL (*p*=0.042, 95% CI = 0.043–2.414), respectively. Blood TAS was lower in the EXG group than in the control group, with a mean difference of 0.262 mmol/L (*p* < 0.0001, 95% CI = −0.393 to −0.132). However, there were no differences in blood TOS and aqueous humor TOS between the EXG group and the control group.

**Conclusions:**

This meta-analysis indicates that OAG patients had a lower TAS in the blood and higher levels of SOD, GPX, and CAT in the aqueous humor, while EXG patients only had a decreased TAS in the blood.

## 1. Introduction

Glaucoma represents a group of diseases defined by characteristic visual dysfunction and optic neuropathy and is a major cause of irreversible blindness worldwide [1]. It has been estimated that the number of people (aged 40–80 years) with glaucoma all over the world was 64.3 million in 2013, increasing to 76.0 million in 2020 and 111.8 million in 2040 [2]. Because of the rapid increase in aging populations worldwide, the prevalence of glaucoma also increased year by year.

The pathologic mechanisms leading to glaucoma are still unclear. Although high intraocular pressure is considered to be the most important risk factor for glaucoma [3], other concomitant factors may also play important roles in the etiology and pathology of the disease, including high glutamate levels [4], alterations in nutritional status [5], vascular factors [6–8], dysfunction of the immune system [9–11], and oxidative stress [12–14]. Growing evidence obtained from clinical and experimental studies over the past decade strongly suggested the involvement of oxidative stress in the degeneration of retinal ganglion cells (RGCs) in glaucoma [14, 15]. Oxidative stress may damage the structure of the trabecular meshwork and increase the resistance to aqueous humor outflow, thus causing the retina to be exposed to ocular hypertension and neurological damage [16]. Progressive neurological damage is followed by RGC death and axon atrophy, which finally lead to irreversible vision loss [17, 18].

Oxidative stress reflects a disbalance between pro-oxidants and antioxidants [19]. In normal conditions, the generation of damaging reactive oxygen species (ROS) is blocked by an antioxidant defense, such as SOD (superoxide dismutase), GPX (glutathione peroxidase), and CAT (catalase). It has been postulated that, in glaucoma, the existence of excess ROS cannot be eliminated by antioxidants effectively. Thus, ROS may damage ocular tissues and produce many small molecules that can be detected both in serum and the aqueous humor. Although the evaluation of general markers of oxidative stress in different types of glaucoma has been assessed in various studies [12, 15, 19, 20], the results are under debate and the study samples were all relatively small. Therefore, we conducted a systematic review and meta-analysis to evaluate oxidative stress status in different types of glaucoma and further investigate the association between oxidative stress and glaucoma.

## 2. Methods

### 2.1. Search Methods

Studies were aggregated from three databases, including PubMed, the Web of Science, and EMBASE. Specifically, randomized controlled trials (RCTs) written in the English language from January 1, 1990, through November 30, 2016, were selected. The following search terms were used to retrieve relevant studies: (glaucoma (Title/Abstract)) AND (total antioxidant (Title/Abstract) OR total oxidant (Title/Abstract) OR malondialdehyde (Title/Abstract) OR superoxide dismutase (Title/Abstract) OR glutathione peroxidase (Title/Abstract) OR catalase (Title/Abstract)). Moreover, a manual search was performed by checking the reference lists of the clinical trials, meta-analyses, and systematic reviews that were examined. Two reviewers (Shengjie Li and Binghua Tang) completed the literature search independently.

### 2.2. Inclusion Criteria

We included studies that met all of the following criteria in the meta-analysis:

1. The investigation involved random sampling or cluster sampling
2. The outcome was suitable for a meta-analysis when subjects and control subjects were measured
3. Cataract subjects/healthy subjects served as the control group
4. The papers were published in the English language
5. It was possible to obtain the full article

**Figure 1:**
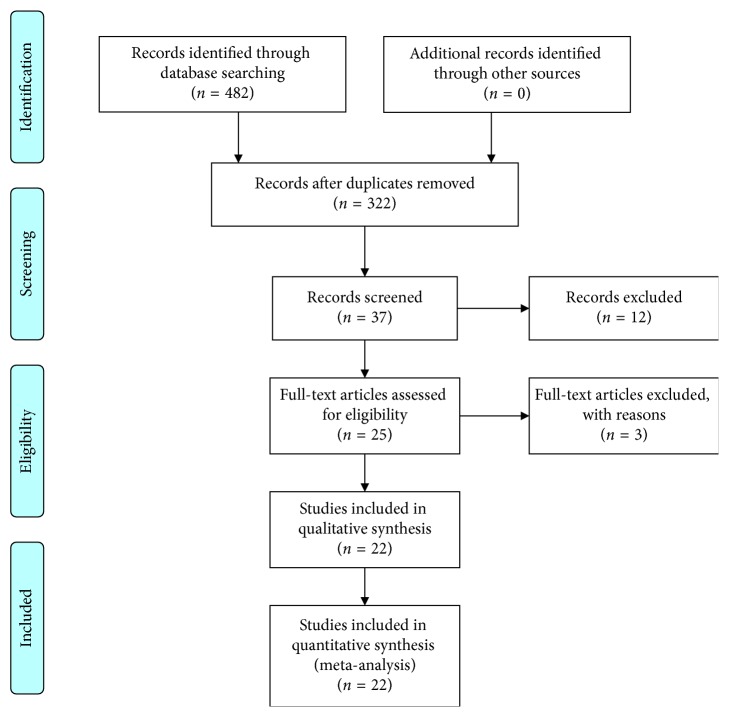
Chart of the article search process.

A flow chart illustrating the article search process is presented in Figure 1.

### 2.3. Data Extraction

We extracted the following data from each study: (1) the name of the first author, (2) the country/region of the study area, (3) the year in which the study was conducted, (4) the age range/mean age of the subjects, (5) the sample size of the study, (6) the method used to sample subjects, (8) the criteria used to diagnose glaucoma, and (9) the required outcome measures. We conducted a focused discussion to resolve any disagreements.

### 2.4. Quality Assessment

The quality assessments of the studies were based on previously reported examination guidelines for glaucoma studies [21, 22]. Five key criteria were used by the two independent investigators to estimate study quality: (1) the sampling scheme, (2) the description of the study population characteristics was adequate, (3) a clear data description was provided, (4) diagnostic criteria for different types of glaucoma, and (5) the detection method of oxidative stress parameters. For each item, studies that were “clear or adequate” scored one point, whereas a “no” scored zero points. A study was considered to be of adequate quality if the quality score was greater than or equal to three. Studies of an inadequate quality were excluded from this meta-analysis.

### 2.5. Statistical Analysis

The statistical analyses were performed using Comprehensive Meta-Analysis version 2.0 (Biostat, Englewood Cliffs, NJ, USA; http://www.meta-analysis.com). The heterogeneity of the pooled studies was estimated using the *χ* ^2^-based *Q* statistic and *I* ^2^ metric. If heterogeneity was observed, a random-effects model was used; otherwise, a fixed-effects model was applied. We conducted direct comparisons using a random-effects model to estimate weighted mean differences and 95% confidence intervals (CIs). A sensitivity analysis, funnel plot analysis, and Egger's test were performed to assess potential publication bias. A value of *p* < 0.05 was considered statistically significant.

## 3. Results

### 3.1. Characteristics of the Studies

A flow chart illustrating the article search process is presented in Figure 1. Using the initial search strategy, we identified 482 articles, and 460 articles were excluded. A total of 22 studies [19, 23–43] were included in this meta-analysis. Detailed characteristics of each included study are presented in Tables 1 [Table tab2] [Table tab3]–4. Twenty-two studies were categorized into four groups according to the type of glaucoma. Specifically, 4 studies were classified in an angle-closure glaucoma (ACG) group, 11 studies in an open-angle glaucoma (OAG) group, 2 studies in a normal tension glaucoma (NTG) group, and 17 studies in an exfoliation glaucoma (EXG) group. Some studies examined multiple types of glaucoma and were placed in multiple groups, which is why more than 22 studies are listed above.

The different sources of specimens (blood or aqueous humor) and the oxidative stress parameters of the studies are as follows:

1. In the 4 studies from the ACG group, 3 sampled blood (total antioxidant status (TAS) 2, MDA 1) and 1 sampled aqueous humor (SOD 1, GPX 1, CAT 1)
2. In the 11 studies from the OAG group, 5 sampled blood (TAS 3, total oxidant status (TOS) 1, MDA 2, SOD 2, CAT 1) and 6 sampled aqueous humors (TAS 3, TOS 1, MDA 2, SOD 4, GPX 4, CAT 4)
3. In the 2 studies from the NTG group, both sampled blood (TAS 2, TOS 1)
4. In the 17 studies from the EXG group, 10 sampled blood (TAS 7, TOS 4, MDA 2, SOD 1, CAT 3) and 7 sampled aqueous humors (TAS 4, TOS 3, SOD 1, GPX 1, CAT 3)

We performed a meta-analysis if the studies examining the same type of glaucoma, the same type of oxidative stress parameters, and the same type of specimens scored greater than or equal to three in our quality assessment. In brief, eight subgroup meta-analyses were performed in this study. The quality of the included trials is shown in Table 5.

### 3.2. Meta-Analysis of the Association of Oxidative Stress Status with OAG

**Figure 2:**
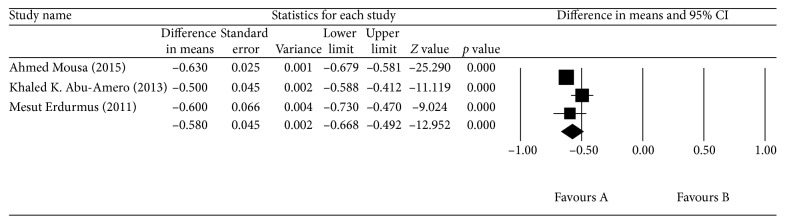
The meta-analysis of blood TAS levels in the OAG group.

**Figure 3:**
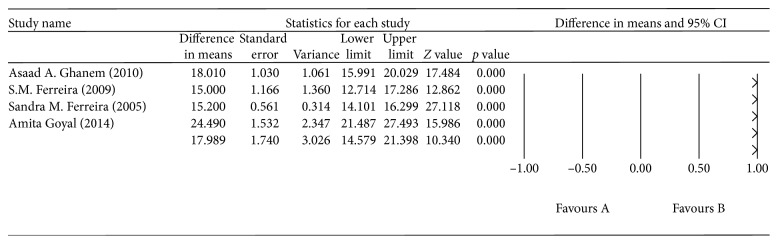
The meta-analysis of aqueous humor SOD levels in the OAG group.

**Figure 4:**
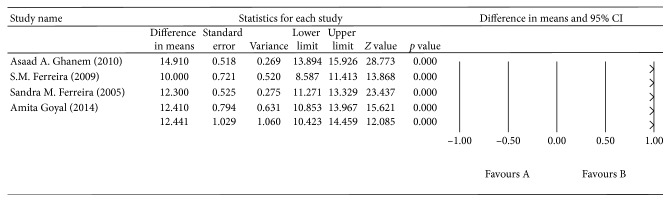
The meta-analysis of aqueous humor GPX levels in the OAG group.

**Figure 5:**
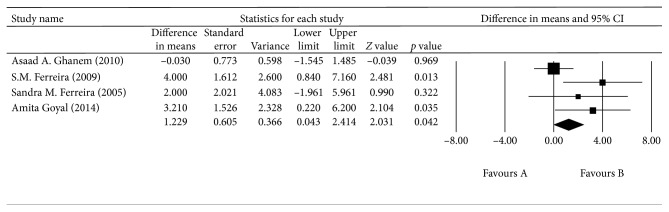
The meta-analysis of aqueous humor CAT levels in the OAG group.

Detailed characteristics of studies in the OAG group are presented in Table 2. The blood TAS was lower in the OAG group than in the control group, with a mean difference of 0.580 mmol/L (*p* < 0.0001, 95% CI = −0.668 to −0.492); however, there was significant heterogeneity across the three studies (*I* ^2^ = 68.725%, *p*=0.041; Figure 2). The aqueous humor SOD levels were higher in the OAG group than in the control group, with a mean difference of 17.989 U/mL (*p* < 0.0001, 95% CI = 14.579–21.298), but, again, there was significant heterogeneity across the four studies (*I* ^2^ = 91.747%, *p* < 0.0001; Figure 3). The aqueous humor GPX levels were higher in the OAG group than in the control group, with a mean difference of 12.441 U/mL (*p* < 0.0001, 95% CI = 10.423–14.459), and there was also significant heterogeneity across the four studies (*I* ^2^ = 90.855%, *p* < 0.0001; Figure 4). The aqueous humor CAT levels were higher in the OAG group than in the control group, with a mean difference of 1.229 fmol/mL (*p*=0.042, 95% CI = 0.043–2.414). There was no significant heterogeneity across the four studies (*I* ^2^ = 59.660%, *p*=0.059; Figure 5).

### 3.3. Meta-Analysis of the Association between Oxidative Stress Status and EXG

**Figure 6:**
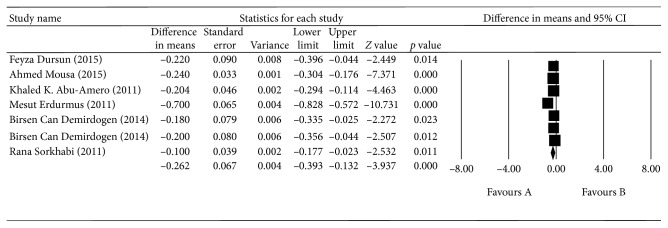
The meta-analysis of blood TAS levels in the EXG group.

**Figure 7:**
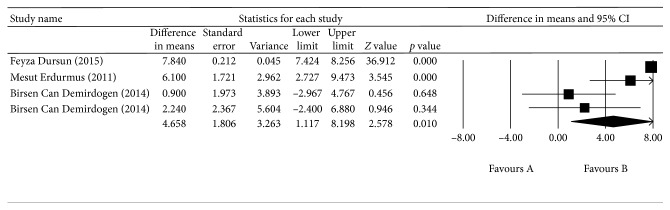
The meta-analysis of blood TOS levels in the EXG group.

**Figure 8:**
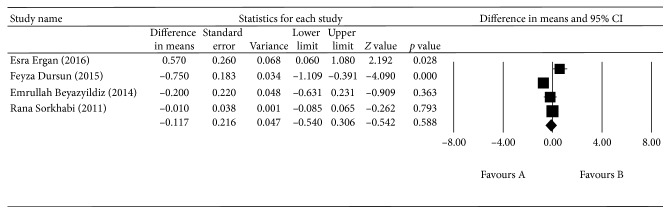
The meta-analysis of aqueous humor TAS levels in the EXG group.

**Figure 9:**
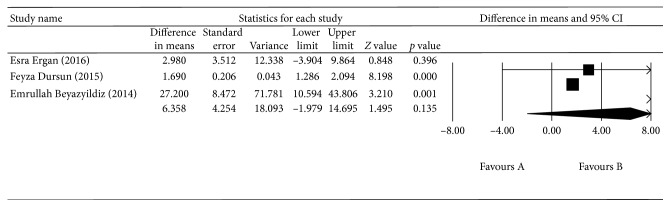
The meta-analysis of aqueous humor TAS levels in the EXG group.

Detailed characteristics of the studies in the EXG group are presented in Table 4. Blood TAS was lower in the EXG group than in the control group, with a mean difference of 0.262 mmol/L (*p* < 0.0001, 95% CI = −0.393 to 0.132). The seven studies were, however, significantly heterogeneous (*I* ^2^ = 90.579%, *p* < 0.0001; Figure 6). There was no difference in blood TOS between the EXG group and the control group (*p*=0.563, 95% CI = −60.657–3.017; Figure 7). There was also no difference in the aqueous humor TAS between the EXG group and the control group (*p*=0.588, 95% CI = −0.540–0.306; Figure 8) or in the aqueous humor TOS between the EXG group and the control group (*p*=0.135, 95% CI = −1.979–14.695; Figure 9).

### 3.4. Sensitivity Analysis and Publication Bias

In the meta-analysis of the association between aqueous humor CAT levels and OAG, a sensitivity analysis revealed that three studies influenced the result. Three studies influenced the meta-analysis results regarding the association between blood TOS and EXG, four studies influenced the results regarding the association between aqueous humor TAS and EXG, and two studies influenced the meta-analysis results regarding the association between aqueous humor TOS and EXG (Table 6). A funnel plot analysis and Egger's test suggested that no publication bias existed in the subgroup analysis (both *p* > 0.05; Supplementary Figures 1–8) except in the blood TAS of the EXG subgroup (*p*=0.015; Supplementary Figure 6).

## 4. Discussion

The pathological mechanisms of OAG and EXG are significantly different, which indicates the specific pathogeneses may also be different. Although oxidative stress is involved in both OAG and EXG, whether it is involved in different types of glaucoma remained to be investigated. In this work, we assessed all studies reporting the level of oxidative and antioxidative markers in aqueous humor or serum samples of glaucoma patients and found that the blood TAS was lower in the OAG group than in the control group, but antioxidant enzymes, including SOD, GPX, and CAT, were increased in the aqueous humors of the OAG group. At the same time, we also found that blood TAS was lower in the EXG group than in the control group, but there was no difference between the blood TOS and the aqueous humor TAS and TOS in the EXG group.

OAG is a multifactorial disease in which aging, genetic factors, inflammation, and oxidative stress may play a specific role [15, 36, 44]. An increased intraocular pressure is a high risk factor of POAG, but normal intraocular pressure can also lead to optic nerve damage and loss of vision in POAG [45]. Although the causes of OAG are unknown, oxidative stress appears to play a role in progressive glaucomatous optic nerve damage [23, 30, 36]. The blood TAS in the meta-analysis of OAG patients decreased, which suggested a breakdown of the balance between the ROS generation and clearance systems. It has been reported that RGCs probably die through an apoptotic process, ultimately leading to glaucomatous optic neuropathy [46, 47], and apoptotic processes are known to involve oxidative stress [48]. Therefore, we speculated that the degeneration of RGCs was induced by oxidative stress. In this meta-analysis, we also found that the activity of SOD, GPX, and CAT in the aqueous humors of OAG patients increased. All of those enzymes have antioxidant effects despite having different specific functions. SOD catalyzes the oxidation/reduction conversion of superoxide radicals to molecular oxygen and hydrogen peroxide [49]. CAT is another key antioxidant enzyme that protects against harmful peroxide by converting hydrogen peroxide to water and oxygen, thereby mitigating its toxic effects [49]. GPX catalyzes the reduction of hydrogen peroxide by two molecules of glutathione as part of an ROS defense system [49]. Therefore, we speculated that the antioxidant enzyme levels increased as a means of resisting the excess ROS in the aqueous humor to avoid their damage to the trabecular meshwork or RGCs. However, there was significant heterogeneity across the studies included in our meta-analysis. The reasons may be that the study population was different, and despite the fact that the same methods were used to detect indicators, the regents or procedures were different.

EXG is an age-related disorder characterized by abnormal synthesis and deposition of extracellular fibrillar material and is the most common cause of secondary OAG [50]. Although the precise mechanisms underlying the development of exfoliative syndrome and the subsequent progression from exfoliative syndrome to EXG remain unclear, a number of studies in the last decade suggest that increased oxidative stress, which leads to exfoliation-induced tissue damage and pathological alterations of the extracellular matrix, is involved in the pathobiology of EXG [20, 34, 37, 38, 40]. The TAS of the EXG group was lower than that of the control group in our meta-analysis, which is consistent with the results that had been reported. It has been shown that oxidative damage in connective tissues such as collagen and elastin can lead to the deposition of extracellular fibrillar [13, 14], which may be the pathogenesis of EXG. However, we did not find significant differences in the blood TOS, aqueous humor TAS, and aqueous humor TOS. The results of other studies that investigated TAS or TOS in aqueous humors remain under debate [20, 24, 38]. The difference may be explained by different study populations and detection methods.

There are several potential limitations of our study. First, we only included studies that scored at least a three in our quality assessment, thus excluding PACG, NTG, and other markers relevant to glaucoma that do not comprehensively demonstrate oxidative stress. Second, most studies evaluated oxidative stress markers in aqueous humors by comparing them to groups with cataracts, which may also involve oxidative stress [51]. Third, our sensitivity analysis revealed some studies influenced the results, especially in the EXG patients. The present data should therefore be interpreted with caution and requires further confirmation in a larger clinical study.

In conclusion, considering the importance of oxidative stress in glaucoma and despite the limitations indicated above, this meta-analysis evaluated all the studies reporting the level of oxidative and antioxidative markers in aqueous humor or serum samples from glaucoma patients, making the results of our study more meaningful. Our study further verified the abnormality of oxidative stress markers in glaucoma. However, reports on the damage of oxidative stress in other eye tissues relevant to glaucoma are still few. Therefore, further research focusing on different aspects will help to provide a comprehensive understanding of oxidative stress in glaucoma. On the other hand, since peripheral blood is easy to collect and has been confirmed to show oxidative stress markers in patients with glaucoma, systemically monitoring the changes in oxidative stress markers may provide new ideas for the prevention and treatment of glaucoma.

## Figures and Tables

**Table 1:** The level of oxidative stress status with ACG.

First author	Year	Country	ACG group							Control group						
			*N*	Age (years)	Total antioxidant status	MDA	SOD	GPX	Catalase	*N*	Age (years)	Total antioxidant status	MDA	SOD	GPX	Catalase
*Blood*																
Ahmed Mousa	2015	Saudi Arabia	139	63.1 ± 9.6	0.98 ± 0.41 (mmol/L)					351	61.8 ± 11.7	1.1 ± 0.22 (mmol/L)				
Khaled K. Abu-Amero	2014	Saudi Arabia	139	63.1 ± 9.4	1 ± 0.22 (mmol/L)					149	61.1 ± 10.8	0.97 ± 0.43 (mmol/L)				
Dong Chang	2011	China	50	59.04 ± 68.42		4.35 ± 0.81 (nmol/ml)				50	60.94 ± 6.95		3.51 ± 0.84 (nmol/ml)			
*Aqueous humor*																
Amita Goyal	2014	India	30				44.38 ± 6.47 U/ml	19.27 ± 3.84 U/ml	37.09 ± 6.60 fmol/ml	30				21.70 ± 4.93 U/ml	8.17 ± 2.97 U/ml	35.57 ± 5.42 fmol/ml

ACG: angle-closure glaucoma; MDA: malondialdehyde; SOD: superoxide dismutase; GPX: glutathione peroxidase.

**Table 2 tab2:** The level of oxidative stress status with OAG.

First author	Year	Country	OAG group								Control group							
			*N*	Age (years)	Total antioxidant status	Total oxidant status	MDA	SOD	GPX	Catalase	*N*	Age (years)	Total antioxidant status	Total oxidant status	MDA	SOD	GPX	Catalase
*Blood*																		
Ugur Yilmaz Mumcu	2016	Turkey	53	58.81 ± 12.23			1.046 ± 0.84 (*μ*mol/L)				52	61.53 ± 10.95			0.47 ± 0.179 (*μ*mol)			
Ahmed Mousa	2015	Saudi Arabia	147	66.8 ± 17.9	0.47 ± 0.32 (mmol/L)						351	61.8 ± 11.7	1.1 ± 0.22 (mmol/L)					
Khaled K. Abu-Amero	2013	Saudi Arabia	139	62.3 ± 11.5	0.47 ± 0.32 (mmol/L)						148	61.1 ± 10.8	0.97 ± 0.43 (mmol/L)					
O. Awodelo	2015	Nigeria	40	57.7 ± 11.2				1.28 ± 1.44 (u/mg protein)		54.8 ± 96.19 (u/mg protein)	20	49.8 ± 20.4				1.95 ± 0.76 (u/mg protein)		56.29 ± 22.01 (u/mg protein)
Mesut Erdurmuş	2011	Turkey	23	62.3 ± 9.0	0.6 ± 0.1 mmol/L	19.6 ± 2.6 *μ*mol/L	1.9 ± 0.2 *μ*mol/L	13 ± 0.5 mg/L			19	57.4 ± 5.6	1.2 ± 0.3 mmol/L	15.1 ± 7.0*μ*mol/L	1.1 ± 0.2 *μ*mol/L	9.4 ± 0.6 mg/L		
*Aqueous humor*																		
Esra Ergan	2016	Turkey	15	67.1 ± 9.6	2.95 ± 2.0 mmol/L	31.59 ± 12.7 (*μ*mol H2O2 Eqv/L)					31	67 ± 9.8	1.9 ± 0.79 mmol/L	28.48 ± 10.08(*μ*mol H2O2 Eqv/L)				
Asaad A. Ghanem	2010	Egypt	30	54.5 ± 4.98			0.48 ± 0.1 *μ*mol/L	39.97 ± 4.82 U/mL	22.63 ± 2.06 U/mL	46.77 ± 3.3 fmol/mL	25	51.28 ± 2.82			0.06 ± 0.02 *μ*mol/L	21.96 ± 1.97 U/mL	7.72 ± 1.72 U/mL	46.8 ± 2.2 fmol/mL
S.M. Ferreira	2009	Argentina	25	70 ± 10				42 ± 5 (U/ml)	16 ± 3 (U/ml)	42 ± 4 (fmol/ml)	25	73 ± 2				27 ± 3 (U/ml)	6 ± 2 (U/ml)	38 ± 7(fmol/ml)
Vicente Zanon Moreno	2008	Spain	50	70.92 ± 8.19	2.818 ± 0.657 mmol/L		0.471 ± 0.134 mmol/L				40	73.75 ± 6.95	4.049 ± 1.577 mmol/L		0.079 ± 0.028 mmol/L			
Sandra M. Ferreira	2005	Argentina	24	71 ± 2	52 ± 7 umol/l Trolox			41.7 ± 2.7 U /ml	18.4 ± 2.5 U/ml	42 ± 7 fmol/ml	24	73 ± 2	124 ± 5 umol/l Trolox			26.5 ± 0.5 U /ml	6.1 ± 0.6 U/ml	40 ± 7 fmol/ml
Amita Goyal	2014	India	30					46.19 ± 6.79 U/ml	20.58 ± 3.18 U/ml	38.78 ± 6.36 fmol/ml	30					21.70 ± 4.93 U/ml	8.17 ± 2.97 U/ml	35.57 ± 5.42 fmol/ml

OAG: open-angle glaucoma; MDA: malondialdehyde; SOD: superoxide dismutase; GPX: glutathione peroxidase.

**Table 3 tab3:** The level of oxidative stress status with NTG.

First author	Year	Country	NTG group				Control group			
			*N*	Age (years)	Total antioxidant status	Total oxidant status	*N*	Age (years)	Total antioxidant status	Total oxidant status
*Blood*										
Necat Yilmaz	2016	Turkey	32	59.6 ± 18.7	2.2 ± 0.32 *μ*mol/L)	3(1.3–7.9) (*μ*mol H_2_O_2_ equivalent/L)	40	54.9 ± 12.5	2.13 ± 0.24 *μ*mol/L)	0.95(0.6–1.2)(*μ*mol H_2_O_2_ equivalent/L)
Kenya Yuki	2010	Japan	43	59.0 ± 10.4	1.170.6 ± 0.0907 (*μ*mol/L)		40	62.2 ± 14.5	1.113.9 ± 0.1031 (umol/L)	

NTG: normal tension glaucoma.

**Table 4:** The level of oxidative stress status with EXG.

First author	Year	Country	EXG group								Control group							
			*N*	Age (years)	Total antioxidant status	Total oxidant status	MDA	SOD	GPX	Catalase	*N*	Age (years)	Total antioxidant status	Total oxidant status	MDA	SOD	GPX	Catalase
*Blood*																		
Feyza Dursun	2015	Turkey	26	66.19 ± 6.88	1.30 ± 0.27mmol/L	8.57 ± 0.95*μ*mol/L					26	67.03 ± 6.34	1.52 ± 0.37(mmol/L)	0.73 ± 0.52*μ*mol/L				
Ahmed Mousa	2015	Saudi Arabia	54	67.6 ± 10.4	0.86 ± 0.24(mmol/L)						351	61.8 ± 11.7	1.1 ± 0.22 (mmol/L)					
Khaled K. Abu-Amero	2011	Saudi Arabia	54	67.57 ± 10.43	0.866 ± 0.241(mmol/l)						54	67.72 ± 10.60	1.07 ± 0.234 (mmol/l)					
Mesut Erdurmuş	2011	Turkey	24	65.7 ± 8.0	0.5 ± 0.1mmol/L	21.2 ± 4.2*μ*mol/L	1.7 ± 0.4*μ*mol/L	11.6 ± 0.2mg/L			19	57.4 ± 5.6	1.2 ± 0.3 mmol/L	15.1 ± 7.0 *μ*mol/L	1.1 ± 0.2 *μ*mol/L	9.4 ± 0.6 mg/L		
George G. Koliakos	2008	Greece	20	70.9 ± 7.9						103 ± 21.4(U/ml)	20	70.8 ± 7.7						189.6 ± 84.3 (U/ml)
George G. Koliakos	2009	Greece	20	71.0 ± 7.5						116 ± 38(U/ml)	20	70.8 ± 7.7						189.6 ± 84.3 (U/ml)
Birsen Can Demirdöğen	2014	Turkey	32	70 (67.0–76.0)	1.90 ± 0.28 (mmol/L)	20.86 ± 7.54 (*μ*mol/L)					32	70.5(62.3–78.3)	2.08 ± 0.35 (mmol Trolox equivalent/L)	19.96 ± 8.23 (*μ*mol/L)				
Birsen Can Demirdöğen	2014	Turkey	30	72 (64.5–77.8)	1.88 ± 0.27 (mmol/L)	22.20 ± 10.35 (*μ*mol/L)					32	70.5(62.3–78.3)	2.08 ± 0.35 (mmol Trolox equivalent/L)	19.96 ± 8.23 (*μ*mol/L)				
Mehmet Tetikoğlu1	2016	Turkey	34	65.53 ± 9.1			28.7 ± 5.7 *μ*mol/L			0.12 ± 0.024 (k/ml)	38	62.34 ± 7.7			30.4 ± 5.3 *μ*mol/L			0.21 ± 0.051 (k/ml)
Rana Sorkhabi	2011	Iran	27	63.89 ± 7.37	0.60 ± 0.15 (mmol/L)						27	65.22 ± 9.79	0.70 ± 0.14 (mmol/L)					
*Aqueous humor*																		
Esra Ergan	2016	Turkey	15	67.1 ± 9.6	2.47 ± 0.9 mmol/L)	31.46 ± 13.2 (*μ*mol /L)					31	67 ± 9.8	1.9 ± 0.79 mmol/L	28.48 ± 10.08 (*μ*mo/L)				
Feyza Dursun	2015	Turkey	26	67.34 ± 6.93	0.80 ± 0.70 mmol/L	2.79 ± 0.67 umol/L					26	67.03 ± 6.34	1.55 ± 0.62 mmol/L	1.10 ± 0.81 (umol/L)				
Emrullah Beyazyıldız	2014	Turkey	17	69.8 ± 8.6	2.3 ± 0.7 mmol/L	57.6 ± 32.4 umol/L					25	68.9 ± 12.0	2.5 ± 0.7 mmol/L	30.4 ± 22.6 umol/L				
S.M. Ferreira	2009	Argentina	25	73 ± 2				44 ± 7 (U/ml)	30 ± 2 (U/ml)	40 ± 5 (fmol/ml)	25	73 ± 2				27 ± 3 (U/ml)	6 ± 2 (U/ml)	38 ± 7 (fmol/ml)
George G. Koliakos	2008	Greece	20	70.9 ± 7.9						10.1 ± 4.5 (U/ml)	20	70.8 ± 7.7						14.6 ± 1.9 (U/ml)
George G. Koliakos	2009	Greece	20	71.0 ± 7.5						12.2 ± 6 (U/ml)	20	70.8 ± 7.7						14.6 ± 1.9 (U/ml)
Rana Sorkhabi	2011	Iran	27	63.89 ± 7.37	0.33 ± 0.13 (mmol/L)						27	65.22 ± 9.79	0.34 ± 0.15 (mmol/L)					

EXG: exfoliative glaucoma; MDA: malondialdehyde; SOD: superoxide dismutase; GPX: glutathione peroxidase.

**Table 5:** Methodological quality of the studies.

Name	Sampling scheme	Population characteristics	Data description	Diagnostic criteria				Methods	Score
				ACG	OAG	NTG	EXG		
Ugur Yilmaz Mumcu	Random stratified sample	Adequate	Clear		Clear 1			HPLC	5
Esra Ergan	Random stratified sample	Adequate	Clear		Clear 2		Clear 3	Colorimetric method	5
Necat Yilmaz	Random stratified sample	Adequate	Clear			Clear 4	Clear 5	Colorimetric measurement method	5
Feyza Dursun	Random stratified sample	Adequate	Clear				No	Colorimetric method	4
Ahmed Mousa	Random stratified sample	Adequate	Clear	European Glaucoma Society guidelines	European Glaucoma Society guidelines		European glaucoma society guidelines	Colorimetric-based assay	5
Emrullah Beyazyıldız	Random stratified sample	Adequate	Clear				No	Colorimetric-based assay	4
Khaled K. Abu-Amero	Random stratified sample	Adequate	Clear	Clear 6				Colorimetric method	5
Khaled K. Abu-Amero	Random stratified sample	Adequate	Clear	Clear 7				Colorimetric-based assay	5
O. Awodelo	Random stratified sample	Adequate	Clear		No			Spectrophotometric method	4
Dong Chang	Random stratified sample	Adequate	Clear	Clear 8				Thiobarbituric acid-reacting substance (TBARS) production	5
Khaled K. Abu-Amero	Random stratified sample	Adequate	Clear				Clear 9	Colorimetric-based assay	5
Mesut Erdurmuş	Random stratified sample	Adequate	Clear		Clear 10		Clear 11	Clear	5
Kenya Yuki	Random stratified sample	Adequate	Clear			Clear 12		Colorimetric method	5
Asaad A. Ghanem	Random stratified sample	Adequate	Clear		Clear 13			Thiobarbituric acid reaction/spectrophotometry	5
S.M. Ferreira	Random stratified sample	Adequate	Clear		Clear 14		Clear 15	Spectrophotometrically	5
George G. Koliakos	Random stratified sample	Adequate	Clear				No	Colorimetric method	4
Vicente Zanon Moreno	Random stratified sample	Adequate	Clear		No			Colorimetric technique	4
S.M. Ferreira	Random stratified sample	Adequate	Clear		Clear 16			Chemiluminescence/spectrophotometrically	5
Birsen Can Demirdöğen	Random stratified sample	Adequate	Clear				Clear 17	Colorimetric method	5
Mehmet Tetikoğlu1	Random stratified sample	Adequate	Clear				Clear 18	Enzymatic analysis	5
Amita Goyal	Random stratified sample	Adequate	Clear	No	No			Spectrophotometrically	4
Rana Sorkhabi	Random stratified sample	Adequate	Clear				Clear 19	Spectrophotometric assay	5

EXG: exfoliative glaucoma; NTG: normal tension glaucoma; OAG: open-angle glaucoma; ACG: angle-closure glaucoma. Clear: (1) we described POAG patients who have intraocular pressure (IOP) higher than 21 mmHg, cup/disk rate 0.3, retinal nerve fiber layer defects in OCT, and visual field defects. (2) Patients >45 years old were diagnosed with PEG if they showed typical pseudoexfoliation material on the lens and/or papillary border with an IOP ≥22 mmHg, cup-to-disc ratio ≥0.3, generalized or partial rim notching on the optic nerve head, peripapillary choroidal atrophy or splinter hemorrhage, and glaucomatous visual field damage according to the Advanced Glaucoma Intervention Study score. Patients with all of these findings, except PEM, were diagnosed with POAG. (3) Patients >45 years old were diagnosed with PEG if they showed typical pseudoexfoliation material (PEM) on the lens and/or papillary border with an IOP ≥22 mmHg, cup-to-disc ratio ≥0.3, generalized or partial rim notching on the optic nerve head, peripapillary choroidal atrophy or splinter hemorrhage, and glaucomatous visual field damage according to the Advanced Glaucoma Intervention Study score. Patients with all of these findings, except PEM, were diagnosed with POAG. (4) Patients with an IOP of 21 mmHg or lower, displaying glaucomatous changes in the optic disc (atrophy, neuroretinal rim loss, and peripapillary hemorrhage) and in the visual field were diagnosed as having NTG. (5) A diagnosis of PEXG was given if the results for the anterior segment of the pseudoexfoliative component showed an IOP exceeding 21 mmHg without typical optic nerve head changes or visual field effects. (6) At least three of the following: (i) clinical documentation of angle closure, defined as the presence of appositional or synnechial closure of the anterior chamber angle involving at least 270° by gonioscopy in either eye; (ii) intraocular pressure elevated to a level ≥21 mmHg measured by Goldmann applanation tonometry; (iii) evidence of characteristic glaucomatous optic disk damage with excavation of the disc causing a cup-to-disk ratio (c/d) vertically of at least 0.70 in at least one eye; and (iv) characteristic peripheral visual field loss including nerve fiber bundle defects (nasal step, arcuate scotoma, and paracentral scotoma) or advanced visual field loss (central and/or temporal island of vision) as tested by using an Humphrey Field analyzer in those patients with vision better than 20/200 or Goldmann Manual perimetry in those with worse vision. (7) (i) appearance of the disc or retinal nerve fiber layer, e.g., thinning or notching of disc rim, progressive changes, nerve fiber layer defect; (ii) the presence of characteristic abnormalities in visual field (e.g., arcuate scotoma, nasal step, paracentral scotoma, and generalized depression) in the absence of other causes or explanation; (iii) age greater than 40 years at the time of recruitment, and (iv) open anterior chamber angles bilaterally on gonioscopy. (8) The diagnostic criteria for PACG were as follows: open and nonoccludable anterior chamber angles with gonioscopy (Volk 3 Mirror Gonio Lens, Mentor, USA), glaucomatous optic disc cupping was identified as a vertical cup-to-disc ratio of optic nerve head 0.6 or more, difference of the vertical cup-to-disc ratio 0.2 or more between both eyes, rim width at superior portion (11–1 h) or inferior portion (5–7 h) of 0.2 or less of disc diameter, or the presence of nerve fiber layer defect. (9) Subjects with PEG were defined as those with clinical evidence of exfoliation material on the pupil margin or anterior lens surface, the presence of glaucomatous optic neuropathy with associated visual field loss in one or both eyes, and documented IOP ≥22 mmHg in either eye. (10) POAG was defined as a progressive optic neuropathy characterized by specific glaucomatous optic nerve head damage and visual field loss associated with elevated IOP. (11) A presence of PEX in PEG is confirmed clinically by small, white deposits of material in the anterior segment, most commonly on the pupillary border and anterior lens capsule. (12) After the diagnosis of primary open-angle glaucoma was made, each patient's IOP was measured at seven time-points over 24 hr (i.e., at 6, 9, 12, 15, 18, 21, and 24 o'clock) with Goldmann applanation tonometry, to identify the patients with normal tension glaucoma. (13) POAG patients with elevated intraocular pressure, correlated visual field loss, and glaucomatous optic nerve head changes criteria. (14) Glaucoma patients included in the study had a diagnosis of POAG or XFG. Structural definition: vertical cup-to-disc ratios (C/D) of 0.7 or more, asymmetry in the C/D of 0.2 or more, and/or thinning of the neuroretinal rim-to-disc ratio of less than 0.1 with corresponding perimetric damage. The Disc Damage Likelihood Scale system was used to evaluate the rim-to-disc ratio. Functional definition: the glaucoma hemifield test outside normal limits, and three adjacent points in the 5% level on the pattern deviation plot, using the 24–2 strategy of the Humphrey perimeter. Visual fields were considered reliable if false-negative and false-positive responses were below 33%. Unreliable visual fields were repeated on the same day. If the second visual field was also unreliable, inclusion was made only on the basis of structural damage. (15) For the diagnosis of XFS, only the presence of the material in the anterior surface of the lens was considered. This surface, with the pupil dilated, was carefully examined for the presence of exfoliative material, using the high magnification of the slit-lamp and adequate illumination. (16) Angles were wide open on gonioscopy. Vertical cup/disk ratio ranged from 0.80 to 0.99, showing severe glaucomatous optic nerve damage. (17) The diagnosis of PEX was made on slit-lamp examination following mydriasis and included the presence of typical pseudoexfoliation material on the anterior lens capsule and/or the pupillary border. PG patients were diagnosed when anterior segment findings of PEX accompanied an IOP > 21 mmHg without treatment, typical optic nerve head changes, and visual field defects. (18) The diagnosis of PEX syndrome was made using a slit-lamp examination after pupillary dilation according to the presence of PEX material on the anterior lens capsule and/or on the pupillary border. (19) PEX syndrome was diagnosed if clinical examination revealed deposition of PEX material on the anterior lens capsule or at the pupillary border, the presence of transillumination defects near the pupil accompanied by normal optic nerve head finding, and intraocular pressure (IOP) less than 21 mmHg.

**Table 6:** Sensitivity analysis using the leave-one-out strategy.

Study omitted	Sample	*Z* value	95% CI	*p* value
OAG (total antioxidant status)	Blood			
Ahmed Mousa (2015)		11.084	−0.633 to −0.433	<0.001
Khaled K. Abu-Amero (2013)		26.848	−0.672 to −0.581	<0.001
Mesut Erdurmus (2011)		8.806	−0.697 to −0.443	<0.001
OAG (SOD)	Aqueous humor			
Asaad A. Ghanem (2010)		5.050	3.059 to 7.021	<0.001
S.M. Ferreira (2009)		5.883	3.620 to 7.236	<0.001
Sandra M. Ferreira (2005)		13.455	3.526 to 4.728	<0.001
Amita Goyal (2014)		5.073	3.242 to 6.293	<0.001
OAG (GPX)	Aqueous humor			
Asaad A. Ghanem (2010)		15.206	10.097 to 13.085	<0.001
S.M. Ferreira (2009)		14.255	11.422 to 15.064	<0.001
Sandra M. Ferreira (2005)		8.121	9.460 to 15.480	<0.001
Amita Goyal (2014)		9.213	9.793 to 15.086	<0.001
OAG (catalase)	Aqueous humor			
Asaad A. Ghanem (2010)		3.311	1.313 to 5.122	0.001
S.M. Ferreira (2009)		1.187	−0.504 to 2.054	0.235^*∗*^
Sandra M. Ferreira (2005)		1.818	−0.090 to 2.396	0.069^*∗*^
Amita Goyal (2014)		1.304	−0.433 to 2.151	0.192^*∗*^
EXG (total antioxidant status)	Blood			
Feyza Dursun (2015)		3.623	−0.414 to −0.123	<0.001
Ahmed Mousa (2015)		3.002	−0.441 to −0.093	0.003
Khaled K. Abu-Amero (2011)		3.338	−0.433 to −0.113	0.001
Mesut Erdurmus (2011)		6.924	−0.241 to −0.135	<0.001
Birsen Can Demirdogen (2014)		3.678	−0.422 to −0.128	<0.001
Birsen Can Demirdogen (2014)		3.630	−0.419 to −0.125	<0.001
Rana Sorkhabi (2011)		3.960	−0.438 to −0.145	<0.001
EXG (total oxidant status)	Blood			
Feyza Dursun (2015)		1.918	−0.072 to 6.578	0.055^*∗*^
Mesut Erdurmus (2011)		1.497	−1.228 to 9.174	0.134^*∗*^
Birsen Can Demirdogen (2014)		1.130	0.209 to 9.006	0.236^*∗*^
Birsen Can Demirdogen (2014)		2.696	1.448 to 9.162	0.007
EXG (total antioxidant status)	Aqueous humor			
Esra Ergan (2016)		1.255	−0.773 to 0.169	0.209^*∗*^
Feyza Dursun (2015)		0.426	−0.256 to 0.398	0.670^*∗*^
Emrullah Beyazyildiz (2014)		0.292	−0.657 to 0.487	0.770^*∗*^
Rana Sorkhabi (2011)		0.387	−0.876 to 0.87	0.699^*∗*^
EXG (total oxidant status)	Aqueous humor			
Esra Ergan (2016)		1.029	−11.808 to 37.886	0.304^*∗*^
Feyza Dursun (2015)		1.151	−9.750 to 37.476	0.250^*∗*^
Emrullah Beyazyildiz (2014)		8.233	1.291 to 2.098	<0.001

OAG: open-angle glaucoma; EXG: exfoliative glaucoma; SOD: superoxide dismutase; GPX: glutathione peroxidase; CI = confidence interval. ^*∗*^The influenced meta-analysis results regarding the association of oxidative stress with OAG and EXG.

## Supplementary Materials

Supplementary Materials Supplementary Figure 1: funnel plot analysis to detect publication bias between the blood TAS level in the OAG group, and the result suggested that no publication bias existed in these studies. Supplementary Figure 2: funnel plot analysis to detect publication bias between aqueous humor SOD levels in the OAG group, and the result suggested that no publication bias existed in these studies. Supplementary Figure 3: funnel plot analysis to detect publication bias between aqueous humor GPX levels in the OAG group, and the result suggested that no publication bias existed in these studies. Supplementary Figure 4: funnel plot analysis to detect publication bias between aqueous humor CAT levels in the OAG group, and the result suggested that no publication bias existed in these studies. Supplementary Figure 5: funnel plot analysis to detect publication bias between blood TAS levels in the EXG group, and the result suggested that there no publication bias existed in these studies. Supplementary Figure 6: funnel plot analysis to detect publication bias between blood TOS levels in the EXG group, and the result suggested that publication bias existed in these studies. We conducted sensitivity analysis using the leave-one-out strategy to remove the publication bias. Supplementary Figure 7: funnel plot analysis to detect publication bias between aqueous humor TAS levels in the EXG group, and the result suggested that no publication bias existed in these studies. Supplementary Figure 8: funnel plot analysis to detect publication bias between aqueous humor TOS levels in the EXG group, and the result suggested that no publication bias existed in these studies.
