# Supplementary material for: The Association of Oxidative Stress Status with Open-Angle Glaucoma and Exfoliation Glaucoma: A Systematic Review and Meta-Analysis
Source: J Ophthalmol. 2019 Jan 15;2019:1803619. doi: 10.1155/2019/1803619 (PMC6350588; doi:10.1155/2019/1803619)
Supplement: Supplementary Materials — Supplementary Figure 1: funnel plot analysis to detect publication bias between the blood TAS level in the OAG group, and the result suggested that no publication bias existed in these studies. Supplementary Figure 2: funnel plot analysis to detect publication bias between aqueous humor SOD levels in the OAG group, and the result suggested that no publication bias existed in these studies. Supplementary Figure 3: funnel plot analysis to detect publication bias between aqueous humor GPX levels in the OAG group, and the result suggested that no publication bias existed in these studies. Supplementary Figure 4: funnel plot analysis to detect publication bias between aqueous humor CAT levels in the OAG group, and the result suggested that no publication bias existed in these studies. Supplementary Figure 5: funnel plot analysis to detect publication bias between blood TAS levels in the EXG group, and the result suggested that there no publication bias existed in these studies. Supplementary Figure 6: funnel plot analysis to detect publication bias between blood TOS levels in the EXG group, and the result suggested that publication bias existed in these studies. We conducted sensitivity analysis using the leave-one-out strategy to remove the publication bias. Supplementary Figure 7: funnel plot analysis to detect publication bias between aqueous humor TAS levels in the EXG group, and the result suggested that no publication bias existed in these studies. Supplementary Figure 8: funnel plot analysis to detect publication bias between aqueous humor TOS levels in the EXG group, and the result suggested that no publication bias existed in these studies. [file 1803619.f1.pdf]

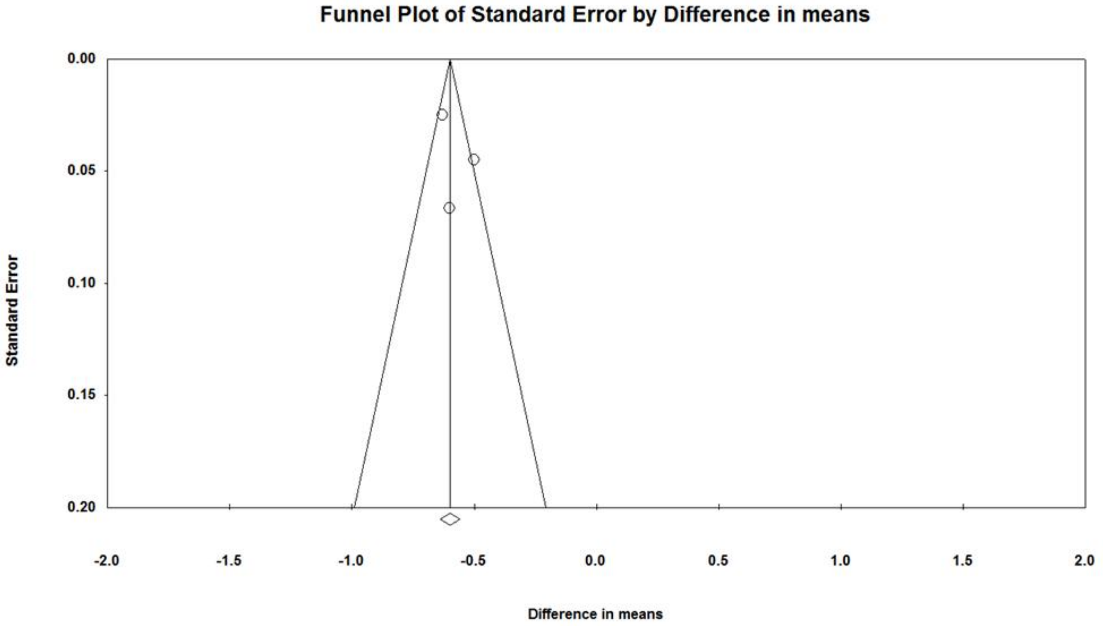

Supplementary figure 1: Funnel plot analysis to detect publication bias between the blood TAS level in the OAG group. And the result suggested that there was no publication bias existed in these studies.

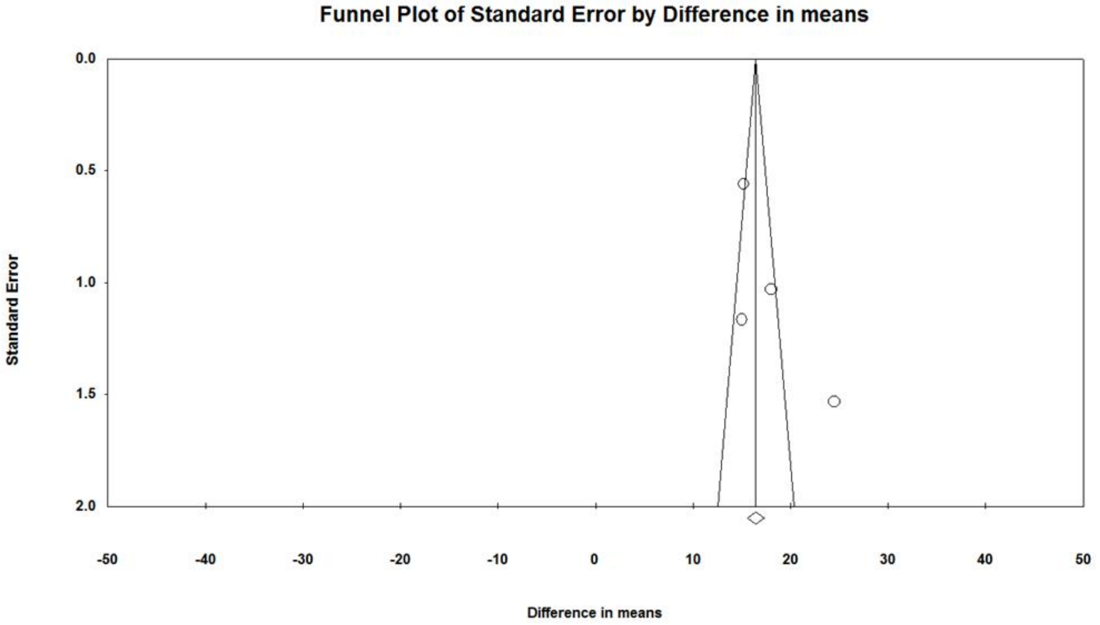

Supplementary figure 2: Funnel plot analysis to detect publication bias between aqueous humor SOD levels in the OAG group. And the result suggested that there was no publication bias existed in these studies.

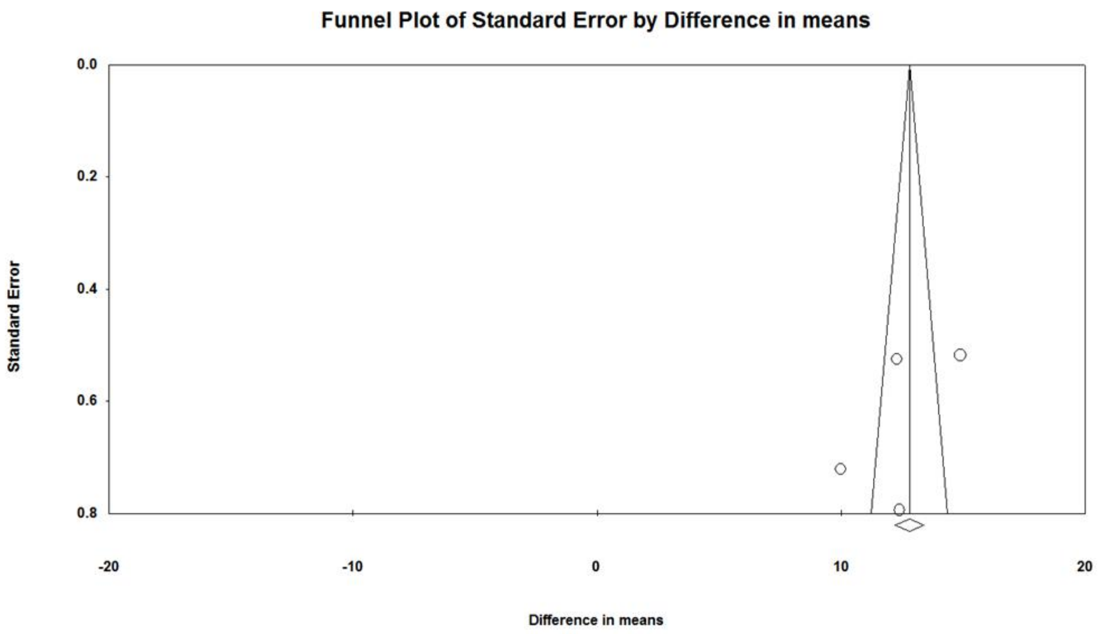

Supplement figure 3: Funnel plot analysis to detect publication bias between aqueous humor GPX levels in the OAG group. And the result suggested that there was no publication bias existed in these studies.

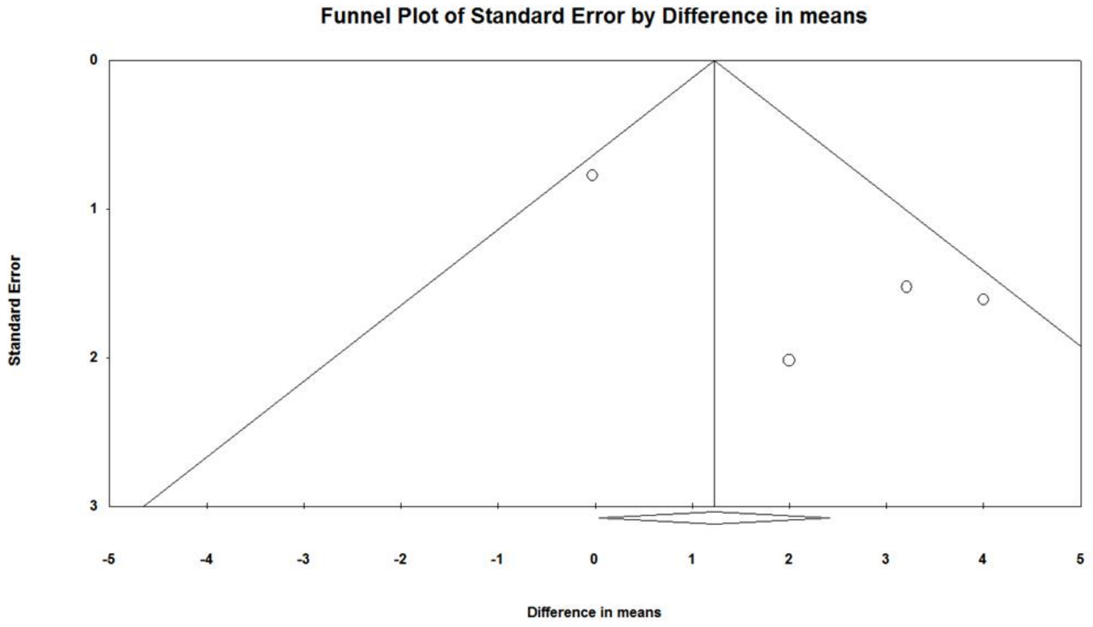

Supplement figure 4: Funnel plot analysis to detect publication bias between aqueous humor CAT levels in the OAG group. And the result suggested that there was no publication bias existed in these studies.

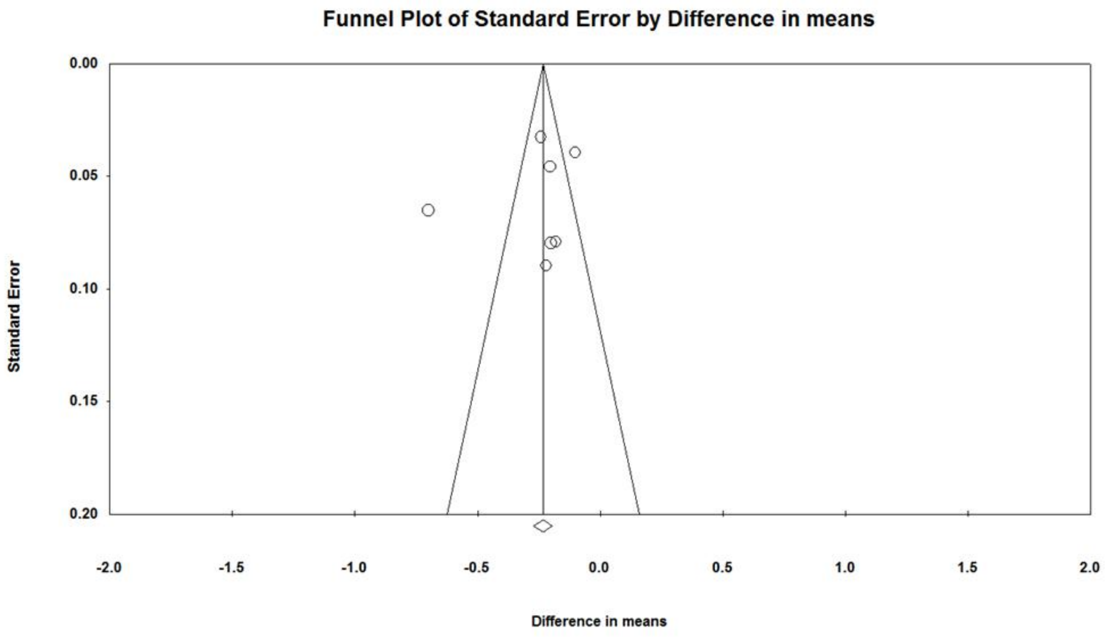

Supplement figure 5: Funnel plot analysis to detect publication bias between blood TAS levels in the EXG group. And the result suggested that there was no publication bias existed in these studies.

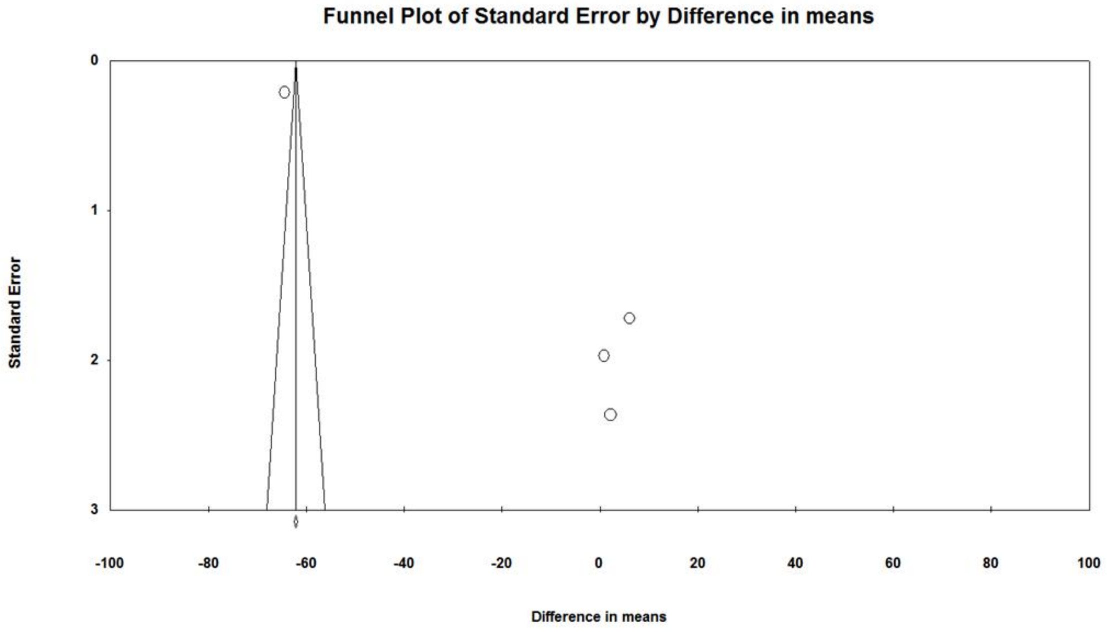

Supplement figure 6: Funnel plot analysis to detect publication bias between blood TOS levels in the EXG group. And the result suggested that there was publication bias existed in these studies. We have make sensitivity analysis using the Leave-one-out Strategy to remove the publication bias.

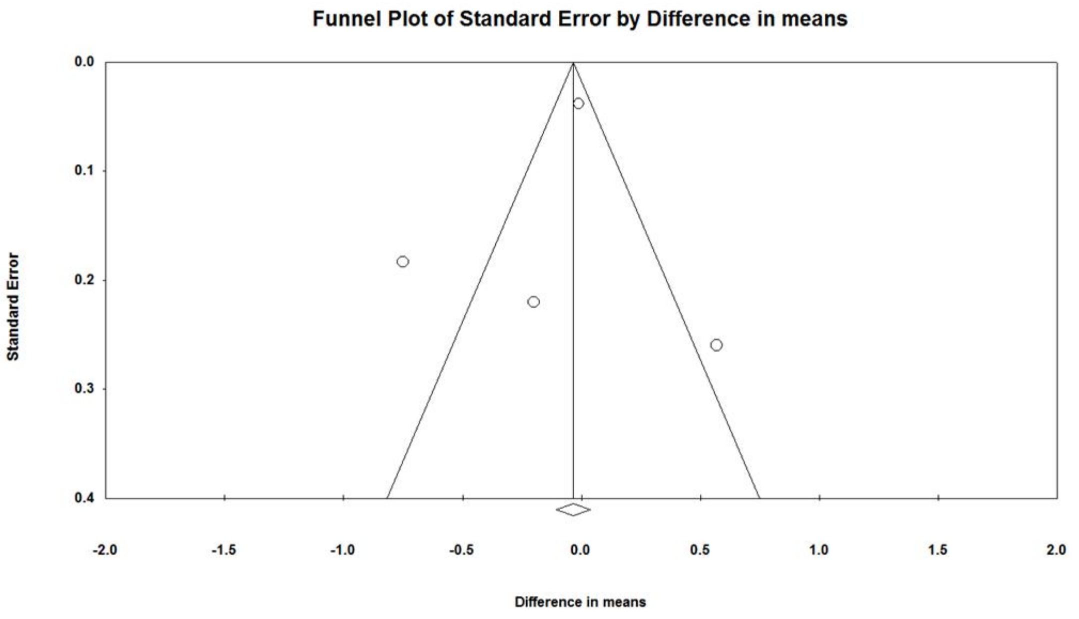

Supplement figure 7: Funnel plot analysis to detect publication bias between aqueous humor TAS levels in the EXG group. And the result suggested that there was no publication bias existed in these studies.

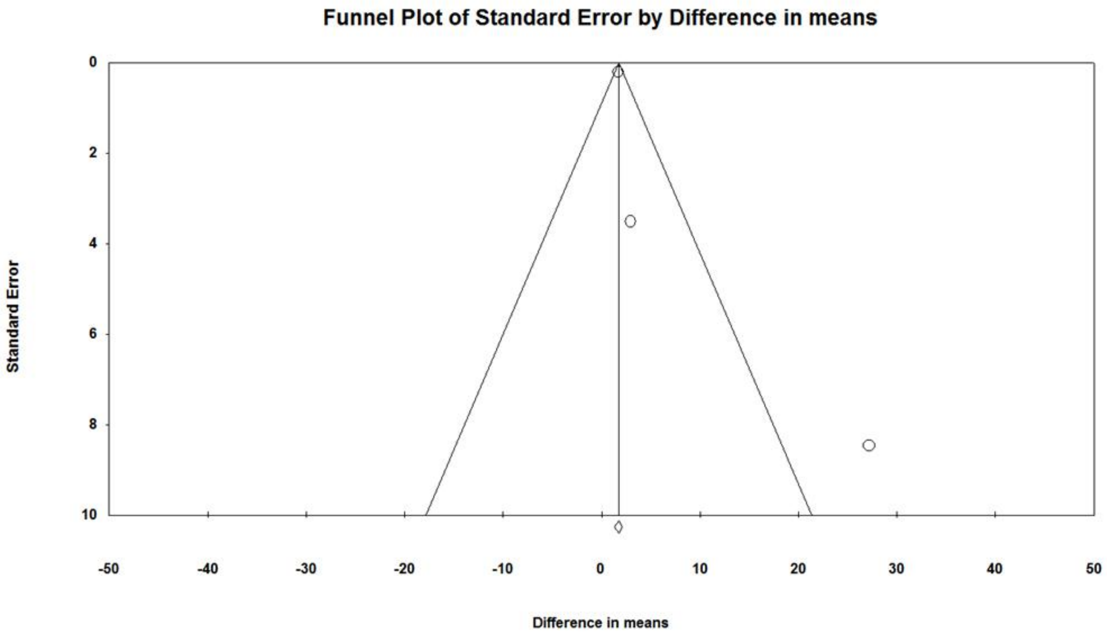

Supplement figure 8: Funnel plot analysis to detect publication bias between aqueous humor TOS levels in the EXG group. And the result suggested that there was no publication bias existed in these studies.
